# Multi-Agent Patrolling under Uncertainty and Threats

**DOI:** 10.1371/journal.pone.0130154

**Published:** 2015-06-18

**Authors:** Shaofei Chen, Feng Wu, Lincheng Shen, Jing Chen, Sarvapali D. Ramchurn

**Affiliations:** 1 College of Mechatronics and Automation, National University of Defense Technology, Changsha, Hunan, 410073, China; 2 School of Electronics and Computer Science, University of Southampton, Southampton, SO171BJ, United Kingdom; 3 School of Computer Science and Technology, University of Science and Technology of China, Hefei, Anhui, 230026, China; Southwest University, CHINA

## Abstract

We investigate a multi-agent patrolling problem where information is distributed alongside threats in environments with uncertainties. Specifically, the information and threat at each location are independently modelled as *multi-state Markov chains*, whose states are not observed until the location is visited by an agent. While agents will obtain information at a location, they may also suffer damage from the threat at that location. Therefore, the goal of the agents is to gather as much information as possible while mitigating the damage incurred. To address this challenge, we formulate the single-agent patrolling problem as a *Partially Observable Markov Decision Process* (POMDP) and propose a computationally efficient algorithm to solve this model. Building upon this, to compute patrols for multiple agents, the single-agent algorithm is extended for each agent with the aim of maximising its marginal contribution to the team. We empirically evaluate our algorithm on problems of multi-agent patrolling and show that it outperforms a baseline algorithm up to 44% for 10 agents and by 21% for 15 agents in large domains.

## Introduction

Unmanned Aerial Vehicles (UAVs) are increasingly becoming essential tools to carry out situational awareness tasks in a number of real-world applications ranging from disaster response [1–3] and security surveillance [3–5]. In these scenarios, multiple UAVs may be deployed to gather information at specific locations as quickly as possible in order to support an ongoing operation. However, such problems are often liable to a high degree of dynamism (e.g., fires may spread, wind direction may change) and uncertainty (e.g., it may not be possible to completely observe the causes of fires or the location of casualties may not be exactly known), and may also contain a number of hazards or threats for the UAVs (e.g., UAVs may fly close to buildings on fire or debris may fall on the UAVs).

In this paper, we consider the scenario where a set of UAVs aim to patrol the area to gather as much information as possible while minimising the negative impact of threats. Crucially, they aim to do so within an environment that is *partially observable* (i.e., the features of the locations are only fully observable where the UAV is located and partially observable at other locations). Hence, when planning the sequence of locations to visit, UAVs have the difficult task of estimating the information to be gained and threats to be encountered at these locations. This problem is compounded by the fact that the dynamism inherent to the environment may cause the information and threats at each location to change over time (i.e., the environment is stochastic). For example, when UAVs visit a building in a disaster area, the building states (intact, about to collapse, collapsing, or collapsed) may correspond to threat states (levels) for UAVs, and the threat at each location may be changing stochastically, such that it switches between “about to collapse” to “collapsed” due to an aftershock [6]. The information in the environment may also change dynamically (e.g., a victim may get out of danger or the fire may get close to a victim).

To date, a number of approaches to information gathering with teams of UAVs have been proposed. However, most of the work [3, 7, 8] focus on developing algorithms for UAVs gathering information in dynamic environments where the model of the features of the environment is *fully observable* and *stationary* (see Related Work section for more details). Furthermore, none of these approaches have considered how threats may affect the information gathering process while the environment is partially observable and non-stationary. Unless such issues are tackled, we believe it is unlikely that large UAV deployments in real major disaster will be feasible.

In recent years, *agent-based modelling* has been effectively used to formulate and solve the problems of planning in environments characterized by uncertainties [9]. In agent-based models, an agent is an encapsulated computer system that is situated in some environment and that is capable of flexible, autonomous action in that environment in order to meet its design objectives [10]. Such agents are either software or hardware (e.g., robots or unmanned autonomous systems (UAS)). In particular, operating in uncertain environments, autonomous agents have to deal with executing actions that may not have the intended results, with environments that change while the agent is operating, and with making observations that might not be completely accurate.

Against this background, we propose a agent-based model for patrolling under uncertainty and threats and go on to develop a novel algorithm to solve the planning problem that it poses. In more detail, we first model the information and threats on a graph representing the environment, where the information and threat at each location are independently modelled as *multi-state Markov chains* (which captures the non-stationary feature), whose states are not observed until the location is visited by an agent (which captures the partially observable feature). Then, we cast the single-agent patrolling problem as a *Partially Observable Markov Decision Process* (POMDP), which provides a rich model for planning and acting in partially observable stochastic domains [11]. Unfortunately, existing POMDP solvers are very inefficient to solve our POMDP formulation due to the exponential growth of the number of possible paths of agents in the size of the graph and the number of the possible observations along each possible path (see Related Work section for more detail). Hence, we propose an online algorithm to solve the patrolling problem for one agent at a time. (In computer science, an online algorithm is one that can process its input piece-by-piece in a serial fashion, i.e., in the order that the input is fed to the algorithm, without having the entire input available from the start. In contrast, an offline algorithm is given the whole problem data from the beginning and is required to output an answer which solves the problem at hand.) In particular, the algorithm utilises a predictive heuristic that only refers to the possible paths (looking ahead several steps) from the current position of the agent. Building upon this, to compute patrolling policies for multiple agents, the single-agent algorithm is extended for each agent with the aim of maximising its marginal contribution to the team. In summary, this paper advances the state of the art in the following ways:

- We propose the first algorithm for multi-agent patrolling under uncertainty and threats. Our formulation does not only capture the partially observable and non-stationary features of the dynamic environment, but also accounts for the health status of the patrolling agents.
- We design a predictive heuristic to estimate the value of each possible path from current position of the agent and provide an online algorithm to solve the patrolling problem for one agent at a time. Moreover, we propose a multi-agent algorithm that sequentially computes policies for individual agents. In particular, we also show that our multi-agent algorithm scale to larger environments (i.e., more than 10 agents) than existing solutions.
- We evaluate our algorithms in simulations and show that our algorithm outperforms a baseline algorithm up to 44% for 10 agents and by 21% for 15 agents.

The remainder of this paper is structured as follows. First, we review the literature on patrolling problems. We then present our model for the problem of multi-agent patrolling under uncertainty and threats. Given this, we formulate the single-agent patrolling problem as a POMDP and provide an algorithm that computes policies for individual agents. Finally, we propose our multi-agent algorithm and evaluate it in the simulations of multi-agent patrolling in a large environment.

## Related Work

In this section, we review related work on agent based model and approaches for multi-agent patrolling problems.

In general, methods to gather situational awareness without considering threats are typically categorised as a class of *information gathering problem*[3], in which agents aim to continuously collect and provide up-to-date situational awareness. For these dynamic environments, previous work [3, 7, 8] consider fully observable (agents can directly observe the underlying state of the environment) stationary models (joint probability distribution of its states do not change when shifted in time). A partially observable model has been proposed in [12], where an agent can only perceive the exact state at its current position. Game-theoretic approaches [13–18] have focused on patrolling to guard important targets in the presence of strategic evaders or intruders; a problem that is characterised by (possibly multiple) attackers attempting to avoid capture or breach a perimeter. The agents’ main challenge in such cases is to detect and capture these attackers in an effort to minimise loss. However, these approaches do not consider the health status of the agents and the damage that agents can suffer while patrolling.

Stationary models of the information/threats are considered in previous work. The work on information gathering in dynamic environments [8] have focused on specific environmental phenomena (e.g., monitoring algal bloom growth in lakes and salt concentration in rivers) rather than stochastic events as in our scenarios. Markov models are widely used to model non-stationary stochastic states in the world, such as the specific ground targets for aircraft [12, 19, 20] and sensors [21], physical activities in wireless network [22], and channel memory in communication systems [23, 24]. However, a number of strict assumptions are made in these works in terms of the Markov models used. For example, each target at each period can be in one of only two states [12, 23] and the matrix of the Markov models must satisfy some special formations [24].

Among these works, a *Markov Decision Process* (MDP) based algorithm that computes policies for individual agents has been proposed in [3] to solve continuous information gathering in fully observable environments. Our formulation in this paper mainly extends [3] to patrolling under threats in partially observable and on-stationary environments and cast the single-agent patrolling problem as a POMDP. However, solving this formulation using current POMDP solvers [25] for all but the smallest instances is impossible due to the exponential growth in the number of possible paths of agents that can be traced in the environment and the number of the possible observations along each path. The POMCP algorithm has been proposed in [26] and has been shown to generate good solution quality and scale to large POMDPs. However, to the best of our knowledge, developing scalable approaches that extend POMCP to solve multi-agent POMDPs is still a open problem. As these possible benchmarks are unable to scale to multi-agent instances of our formulation, we design a baseline algorithm that greedily select the policy for one time step as a benchmark.

## Methods

In this section, we present the model for the problem of multi-agent patrolling under uncertainty and threats. Specifically, we first model the physical environment in which the agents operate and then go on to describe the decision problem faced by the agents.

### The Patrolling Problem

We formulate the patrolling problem by defining the physical environment and patrolling agents. In particular, we present the Markov models of information and threat at in the environment to capture the non-stationary feature.

#### The physical environment

The *physical environment* is defined by its spatial, temporal and dynamic properties. In particular, in the aftermath of a disaster, a number of specific sites might need urgent attention and access to these sites may be limited to certain areas (e.g., due to trees, debris, or natural obstacles). Hence, we can capture such features in terms of paths along which agents can travel from one disaster site to another. Specifically, the spatial property of the environment is encoded by a graph, which specifies how and where agents can move.

**Definition 1 (Graph)** *We model an area of the environment as an undirected graph G = (V,E), where each vertex V representing spatial coordinates are embedded in Euclidean space and edges E encode the movements that are possible between them. Here, we denote N = ∣V∣.*

In disaster response, each disaster site is a vertex in the graph, and a traversable area between a pair of sites is an edge of the graph.

**Definition 2 (Time)** *Time is modelled by a set of time steps {1,2,…,T} and at each time step t ∈ {1,2,…,T} the agents visit some sites in the environment.*

To capture the dynamic attributes of the environment, we assume that each vertex holds two states: one for information and one for threats.

**Definition 3 (Information State Variable)** *An information state variable indicates different levels of the information at a given vertex.*

For example, how many people need help and what is the status of a bridge are information state variables in disaster response scenario.

**Definition 4 (Threat State Variable)** *A threat state variable reflects the level of damage an agent suffers when visiting a given vertex.*

For example, the level of fire and the degree of smog are typical threat state variables in disaster response.

**Definition 5 (Markov Model of Information and Threat)** *The two state variables at each vertex change over time according to discrete-time multi-state Markov chains.*

**Fig 1:**
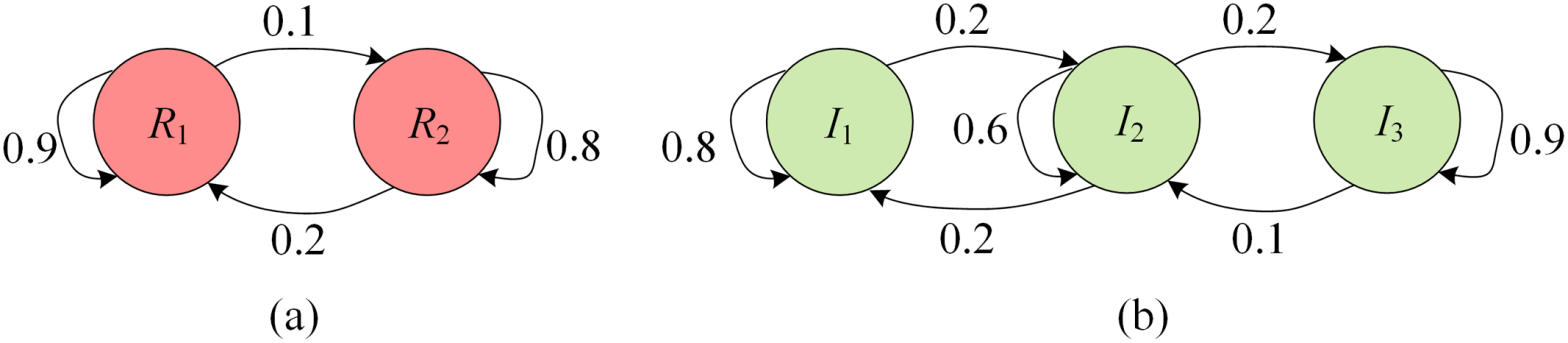
Example of information and threat models at a vertex. (a) A threat model with 2 states (i.e., *R*_1_ and *R*_2_) and (b) an information model with 3 states (i.e., *I*_1_, *I*_2_ and *I*_3_), where the probabilities of each information/threat state changes to another over a time step are given (e.g., the probability of *R*_1_ changes to *R*_2_ is 0.1).

To capture the transitions of the state variables, we employ a Markov chain model. Specifically, for a Markov chain with *K* states *S* = (*S*_1_, *S*_2_, …, *S*_*K*_), the matrix of transition probabilities for pairs of states is defined as:

$$\begin{array}{c} P=[\begin{array}{ccc} p_{11} & \cdots & p_{1K}\\ p_{21} & \cdots & p_{2K}\\ \vdots & \ddots & \vdots \\ p_{K1} & \cdots & p_{KK} \end{array}]=[\begin{array}{c} P_{1}\\ P_{2}\\ \vdots \\ P_{K} \end{array}] \end{array}$$

where *p*_*ij*_ is the probability that threat state *S*_*i*_ transitions to *S*_*j*_ in one time step and *S*_*i*_, *S*_*j*_ ∈ *S*. An example of information and threat models at a vertex is shown in Fig 1. Thus, Fig 1(a) shows a threat model with 2 states (i.e., *R*_1_ and *R*_2_) and Fig 1(b) shows an information model with 3 states (i.e., *I*_1_, *I*_2_ and *I*_3_), where the probabilities of each information/threat state changes over a time step are given (e.g., the probability of *R*_1_ changes to *R*_2_ is 0.1).

The set of information states $I^{n}=\{I_{1}^{n},I_{2}^{n},\ldots ,I_{K_{I}^{n}}^{n}\}$ for location *v*_*n*_ correspond to an amount $K_{I}^{n}$ of information which agents obtain when visiting *v*_*n*_. The value of information is determined by the function *f* ^*n*^:*I* ^*n*^ → ℝ^+^, and $f(I_{k}^{n})$ increases monotonically with $k\in \{1,\ldots ,K_{I}^{n}\}$, which indicates that the states of information are ordered in terms of their value. The information state at a given vertex independently evolves as a $K_{I}^{n}$-state Markov chain model with a matrix of transition probabilities $P_{I}^{n}$.

Similarly, the set of threat states $R^{n}=\{R_{1}^{n},R_{2}^{n},\ldots ,R_{K_{R}^{n}}^{n}\}$ indicate the $K_{R}^{n}$ threat levels of vertex *v*_*n*_ ∈ *V*. The “damage” that an agent suffers when visiting vertex *v*_*n*_ is captured by the function *c* ^*n*^:*R* ^*n*^ → ℝ^+^, and $c(R_{k}^{n})$ increases monotonically with $k\in \{1,\ldots ,K_{R}^{n}\}$. The threat state at a given vertex independently evolves over time as a $K_{R}^{n}$-state Markov chain and the matrix of transition probabilities is $P_{R}^{n}$.

Having modelled the environment in which the agents operate, we next elaborate on the agents’ goals.

#### Patrolling Agents

We define a *patrolling agent* (agent for short) as a physical mobile entity situated in the environment defined above, capable of gathering information, and maybe damaged by the threat when visiting a vertex. The set of all agents is denoted as *A* = {1,…,∣*A*∣}. Then, the movement and visit capabilities of agents are formulated as follows. When patrolling in a graph *G*, each agent is positioned at a given vertex in *G* at each time step *t*. The movement of each agent is atomic, i.e., takes place within the interval between two subsequent time steps, and is constrained by *G*, i.e., agent *m* positioned at a vertex *v*_*i*_ ∈ *V* can only move to a vertex $v_{i}^{\prime }\in adj_{G}(v_{i})$ that is adjacent to *v*_*i*_ in *G*. We assume that ∀*v*_*i*_ ∈ *V*, *v*_*i*_ ∈ *adj*_*G*_(*v*_*i*_), i.e., an agent can also stay at the same vertex. The speed of the agents is sufficient to reach an adjacent vertex within a single time step. Time can be discretised according to the speed of the UAVs. Thus if the UAVs can travel between sites in a five minutes, then a time step may be set at 5 minutes in the model.

Given this, an agent visits a vertex *v*_*n*_ when it is positioned at that vertex. On the one hand, a visit results in the agent being aware of the current information and threat state at *v*_*n*_, such as $I_{i}^{n}$ and $R_{j}^{n}$ respectively. On the other hand, this agent obtains a reward $f^{n}(I_{i}^{n})$ and suffers a loss $c^{n}(R_{j}^{n})$ for a visit. The time it takes to visit a vertex is assumed to be negligible. We let $F^{n}=[f^{n}(I_{1}^{n}),\ldots ,f^{n}(I_{K_{I}^{n}}^{n})]$ denote the information value vector, where *f* ^*n*^(*I*_*k*_) is the information value that an agent could get if the information state is $I_{k}^{n}$ (e.g., information at a vertex has 3 states and corresponds to 3 information values [0, 2, 5]). Similarly, we let $C^{n}=[c^{n}(R_{1}^{n}),\ldots ,c^{n}(R_{K_{R}^{n}}^{n})]$ denote as the damage value vector at vertex *v*_*n*_, where $c^{n}(R_{k}^{n})$ is the damage value that an agent will lose if the threat state is $R_{k}^{n}$ (e.g., fire level at a position has 4 states which corresponds to 4 levels of damage [0, 4, 6, 10], and smog degree at a position has 3 states which corresponds to 4 levels of damage [0, 2, 5]). For each visit, the information at that vertex is obtained by the agent and we regard that the information state at a given vertex *v*_*n*_ will reset to $I_{1}^{n}$ when an agent visits this vertex ($I_{1}^{n}$ is the information state which means no new information was generated at *v*_*n*_ after last visit). As the states at each vertex change over time and agents can only access the exact states at the vertices that they visit, the patrolling environment can be considered non-stationary (i.e., joint probability distribution of its states may change when shifted in time) and partially observable.

Furthermore, in this paper, we make two assumptions about the communication and cooperation among agents as follows.

**Assumption 1** *All the agents can share their collected observations with each other via communication. Such peer to peer communication is free of noise, costs, and delays.*

Consider a centralised station is organized to coordinate a team of UAVs for monitoring the continuously changing state of a disaster area, where each UAV can full communication with this station and Assumption 1 is satisfied in these domains. However, in some real scenarios, UAVs can only coordinate with each other using limited communication and decentralised approaches may be more appropriate (but this is beyond the scope of this paper and will be considered in future work).

**Assumption 2** *When more than one agent is visiting a vertex, only one information value is obtained for the team but each agent suffers the same damage that may be generated at that vertex.*

This assumption is satisfied in the scenarios where the information gathering capability of one agent at a vertex is equal to that provided by multiple agents with the same sensors, and agents independently suffer the damage caused by threats. In future work, a model of information fusion for multiple (heterogeneous) agents will be considered. Thus, the team of agents need to coordinate with each other based on their observations while patrolling. Specifically, the goal of the agents is to gather as much information as possible while minimise the damage incurred.

We now provide a simple example to explain how the agents would operate in this scenario. Consider an agent that enters into a building on fire. In our setting, this is equivalent to the agent visiting a node in the graph. The fire level (threat state variable) and valuable information about victims and assets (information variable) changes over time. While exploring the building, the agent may acquire some information and suffer some damage due to the fire. At each time step, an agent selects one adjacent building to visit based on the estimated information value and the prior observation of threat states at each location. It then obtains a reward based on the value of the information, and suffers a loss which is associated with the threat state. Then, the information state at the visited vertex is immediately reset.

Having defined the patrolling problem, we now need to plan the sequential patrolling actions for agents based on the history of actions and observations, and the model of the environment. Hence, in what follows, we first propose a POMDP formulation for single-agent patrolling within a graph and design an algorithm to solve it. Building upon this, we propose a scalable multi-agent patrolling algorithm.

### Single-Agent Patrolling

In this section, we first formulate the POMDP based framework for single-agent patrolling problem. POMDPs imply that the agent does not know the exact state it is in, and the agent requires to keep track of each observation received, in order to maintain a probability distribution, known as the belief state, over the possible states [11]. Thus, we analysis that a standard representation of belief state makes the POMDP computational intractable and then present a compact representation of belief state for our POMDP formulation. Given this, we propose a predictive heuristic and an online single-agent algorithm.

#### The POMDP Framework

We now set up the single-agent patrolling problem as a POMDP ⟨𝓢,𝓐,𝓞, *T*,Ω, *r*⟩ as follows:

- 𝓢 is the set of states. A *state* is defined as a tuple $s=[v,(s_{R}^{1},\ldots ,s_{R}^{N}),(s_{I}^{1},\ldots ,s_{I}^{N})]\;\in \;𝓢$, where *v* is the current position of the agent, $s_{R}^{N}\;\in \;R^{n}$ and $s_{I}^{N}\;\in \;I^{n}$ are the threat and information states at vertex *v*_*n*_ ∈ *V*. We denote $s_{e}\;\;=\;\;[(s_{R}^{1},\ldots ,s_{R}^{N}),(s_{I}^{1},\ldots ,s_{I}^{N})]\;\in \;{𝓢}_{e}$, as the state that captures the information and threat states at all of the positions. Given this, the number of the states in 𝓢 increases exponentially with the number the vertices.
- 𝓐 is the set of all actions. The agent select an adjacent vertex to visit as an *action*.
- 𝓞 is the set of observations. We define an *observation* $o=(v_{i},s_{I}^{i},s_{R}^{i})\in 𝓞$ as the current position *v*_*i*_ and the information and threat state at this position.
- 𝓣 is the set of conditional transition probabilities. We assume that *v* is deterministic and only determined by the destination of movement of the agent. Based on the Markov models defined in the patrolling problem, *s*_*e*_ follows a discrete-time Markov process with $\prod_{n=1}^{N}K_{R}^{n}K_{I}^{n}$ states.
- Ω is the set of observation probabilities. As an observation *o* is directly a part of some states, the observation probability Ω(*o*∣*s*′, *a*) = 1 if *o* is consistent with the corresponding part of *s*′ and Ω(*o*∣*s*′, *a*) = 0 otherwise.
- *r*:𝓐 × 𝓞 → ℝ is a reward function. *r*(*a*, *o*) is the sum of the rewards obtained by the agent which associates to the action *a* and observation *o*:
  $$\begin{array}{c} r\left(a,o\right)=\alpha f^{i}\left(s_{I}^{i}\right)-\left(1-\alpha \right)c^{i}\left(s_{R}^{i}\right) \end{array}$$ (1)
  where *α* is a weight parameter of the two objectives.

The objective of the agent is then to choose the movement actions sequentially to maximize the total expected reward accumulated over *T* steps.

In this model, the states are not directly observable. Hence, a standard belief vector *B*(*t*) = [*b*_1_(*t*), …, *b*_*M*_(*t*)] is defined as the posterior probability distribution over the possible states *S*, where *b*_*m*_(*t*) is the conditional probability that the environment state is at the *m*th state at the current time step *t*. For any *t*, it has been shown in [27] that this belief vector is a sufficient statistic for the design of the optimal action for each time step. A policy *π* specifies the action that will be executed in any given belief state and the optimal policy π* is a policy by which the agent gets the maximum total expected reward accumulated over *T* steps. However, as each environment state is an joint state of the information and threat states at all of the vertices, the number of possible states *S* that defined in our POMDP is $\prod_{n=1}^{N}K_{R}^{n}K_{I}^{n}$, which increases exponentially with the number the vertices. Moreover, as the belief vector is defined as the posterior probability distribution over these possible states, the dimension of this belief vector also increases exponentially with the number the vertices.

To address this, we propose an online method by introducing a belief vector of reduced dimension and develop a predictive heuristic to reduce the search space and still produce high quality solutions (as we show later).

#### Compact Belief Representation

As the threat state and information state variables at each vertex evolve independently and *v* is deterministic, we can find a sufficient statistic for the optimal policy whose dimension linearly grows with *N*, similar to [23, 24]. We introduce a compact representation of belief state and its transition function in this section.

We define a sufficient statistic belief vector of the environment states at time *t* as the vector of the conditional probabilities (conditioned on the observation and decision history) Ψ(*t*) = [Ψ_*R*_(*t*),Ψ_*I*_(*t*)], where Ψ_*R*_(*t*) is defined as:

$$\begin{array}{c} \left\{ \begin{array}{ccc} \Psi_{R}\left(t\right) & = & \left(w_{R}^{1}\left(t\right),\ldots ,w_{R}^{N}\left(t\right)\right)\\ w_{R}^{n}\left(t\right) & = & \left(w_{R1}^{n}\left(t\right),\ldots ,w_{RK_{R}^{n}}^{n}\left(t\right)\right) \end{array}\right. \end{array}$$ (2)

where $w_{Rk_{1}}^{n}(t)\;$ is the probability that the threat state at vertex *v*_*n*_ is $R_{k_{1}}^{n}\;$, $k_{1}=1,\ldots ,K_{R}^{n}$ and Ψ_*I*_(*t*) is defined as:

$$\begin{array}{c} \left\{ \begin{array}{ccc} \Psi_{I}\left(t\right) & = & \left(w_{I}^{1}\left(t\right),\ldots ,w_{I}^{N}\left(t\right)\right)\\ w_{I}^{n}\left(t\right) & = & \left(w_{I1}^{n}\left(t\right),\ldots ,w_{IK_{I}^{n}}^{n}\left(t\right)\right) \end{array}\right. \end{array}$$ (3)

where $w_{Ik_{2}}^{n}(t)\;$ is the probability that the information state at vertex *v*_*n*_ is $I_{k_{2}}^{n}$ and $k_{2}=1,\ldots ,K_{I}^{n}$. Then Ψ(*t*) is a sufficient statistic of optimal decision making [23, 24]. By exploiting the statistical independence among vertices, we reduce the dimension of the sufficient statistic from $\prod_{n=1}^{N}K_{R}^{n}K_{I}^{n}$ to $\Sigma_{n=1}^{N}(K_{R}^{n}+K_{I}^{n})$, which grows with *N* linearly. This allows us to reduce the computational and storage complexity of the optimal patrolling policy from exponential to linear.

**Theorem 1** *For any time t, Ψ(t) is a sufficient statistic for the design of optimal policy for our POMDP formulation.*

**Proof** We show that when the information and threat at the *N* vertices evolve independently, each element *b*_*m*_(*t*) in the standard belief vector *B*(*t*) can be obtained from Ψ(*t*), where *b*_*m*_(*t*) is the conditional probability that the environment state is at the *i*th state. Without loss of generality, we consider *N* = 2. Let ℐ(*t*) denote the history up to the beginning of slot *t*. Let *τ*_*n*_ denote the most recent time instant when vertex *v*_*n*_ is visited. We can thus write an entry of *b*_*m*_(*t*) as in Eq (4). Quantities in Eq (4) are entries of Ψ(*t*). Hence, Ψ(*t*) is a sufficient statistics.

$$\begin{array}{c} \begin{array}{cc} & \mathrm{Pr}[s_{R}^{1}\left(t\right)\;=\;i^{\prime },s_{R}^{1}\left(t\right)\;=\;i^{\prime \prime },s_{I}^{1}\left(t\right)\;=\;j^{\prime },s_{I}^{2}\left(t\right)\;=\;j^{\prime \prime }|𝓘\left(t\right)]\\ \;=\; & \mathrm{Pr}[s_{R}^{1}\left(t\right)\;=\;i^{\prime },s_{R}^{2}\left(t\right)\;=\;i^{\prime \prime },s_{I}^{1}\left(t\right)\;=\;j^{\prime },s_{I}^{2}\left(t\right)\;=\;j^{\prime \prime }|s_{R}^{1}\left(\tau_{1}\right)\;=\;o_{R}^{{}^{\prime }},s_{R}^{2}\left(\tau_{2}\right)\;=\;o_{R}^{{}^{\prime \prime }},\tau_{1},\tau_{2}]\\ \;=\; & \mathrm{Pr}[s_{R}^{1}\left(t\right)\;=\;i^{\prime }|s_{R}^{1}\left(\tau_{1}\right)\;=\;o_{R}^{{}^{\prime }}]\mathrm{Pr}[s_{R}^{2}\left(t\right)\;=\;i^{\prime \prime }|s_{R}^{2}\left(\tau_{2}\right)\;=\;o_{R}^{{}^{\prime \prime }}]\\ & \mathrm{Pr}[s_{I}^{1}\left(t\right)\;=\;j^{\prime }|\tau_{1}]\mathrm{Pr}[s_{I}^{2}\left(t\right)\;=\;j^{\prime \prime }|\tau_{2}] \end{array} \end{array}$$ (4)

Initially, we assume that we have probabilistic information about the state of each of the *N* vertices Ψ(0) = [Ψ_*R*_(0),Ψ_*I*_(0)]. Then, the elements of belief vector Ψ(*t*) are updated to Ψ(*t*+1) upon action *a* = *v*_*i*_ and observation $o=(v_{i},s_{I}^{i},s_{R}^{i})$ as:

$$\begin{array}{c} \begin{array}{c} w_{R}^{n}\left(t+1\right)=\{\begin{array}{cc} {\tilde{I}}_{k} & \text{if}\;v_{n}=v_{i},s_{R}^{i}\left(t\right)=R_{k}\\ w_{R}^{n}\left(t\right)P_{R}^{n} & \text{if}\;v_{n}\neq v_{i} \end{array}\\ w_{I}^{n}\left(t+1\right)=\{\begin{array}{cc} {\tilde{I}}_{1} & \text{if}\;v_{n}=v_{i}\\ w_{I}^{n}\left(t\right)P_{I}^{n} & \text{if}\;v_{n}\neq v_{i} \end{array} \end{array} \end{array}$$ (5)

where ∀*v*_*n*_ ∈ *V*, $R_{k}^{n}\in R^{n}$, $I_{k}^{n}\in I^{n}$, and ${\tilde{I}}_{k}$ is a unit vector with the *k* ^th^ item is 1, $P_{R}^{n}$ and $P_{I}^{n}$ are respectively the matrices of transition probabilities of threat and information at position *v*_*n*_. As shown in Eq (5), the threat belief vector $w_{R}^{n}(t+1)$ at one vertex *v*_*n*_ that some agent is visiting is updated to ${\tilde{I}}_{k}\;$ based on the observation $R_{k}^{n}(h)$ at this vertex, while ${\text{w}}_{R}^{n}(t+1)\;$ at some other vertex that no agent is visiting is updated by the current threat belief vectors ${\text{w}}_{R}^{n}(t)\;$ and threat Markov model $P_{R}^{n}$ at this vertex. A similar explanation holds for the update to the information belief $w_{I}^{n}(t+1)\;$, as for *v*_*n*_ ∈ *V*.

Based on the transition function above, a policy *π* specifies a sequence of actions *π* = [*π*(1), *π*(2),…], where *π*(*t*) is the position selected to visit at time *t*. Given this, the optimal policy can be computed as:

$$\begin{array}{c} \pi^{*}=\underset{\pi }{\text{arg max}}\;{𝔼}^{\pi }[\sum_{t=1}^{\infty }\gamma^{t}{𝓡}^{\pi (t)}(\Psi (t)|\Psi (0))] \end{array}$$ (6)

where ℛ^*π*(*t*)^(Ψ(*t*)) is the reward obtained when the belief state is Ψ(*t*) and *γ* ∈ [0, 1] is the discount factor.

Although the dimensionality of the belief state is reduced, the problem is still a POMDP and finding the optimal solution is intractable. Based on this reduced belief vector, we next develop a predictive heuristic and present the online single-agent algorithm that implements this heuristic.

#### The Predictive Heuristic

In order to develop a predictive heuristic for online policy selection, we first introduce the assumption that the Markov state transition matrices are monotone matrices, which means that the higher the information/damage value of the vertex’s current state the higher is the likelihood that the next state of this vertex will be of high information/damage value. Then, we show how to define the predictive heuristic as the predictive expected future reward based on the monotonicity of the transition matrices.

Stochastic dominance is a central theme in a wide variety of applications in economics, finance and statistics [28]. Similar assumption has been made to model the states of the channels in communication systems [23, 24] and the states at targets for UAVs monitoring [12]. Stochastic dominance ≻ between two *Z* dimension probability vector *x*, *y* is defined as *x* ≻ *y*, if:

$$\begin{array}{c} \sum_{j=i}^{Z}x\left(j\right)\geq \sum_{j=i}^{Z}y\left(j\right),\;\text{for}\;i=2,3,\ldots ,Z \end{array}$$ (7)

We assume that the Markov information model and Markov threat model are monotonic matrices, i.e., the matrix of transition probabilities $P_{R}^{n}$ and $P_{I}^{n}$ satisfies:

$$\begin{array}{c} \begin{array}{cccc} P_{RK_{1}}^{n} & ≻ & P_{RK_{1}-1}^{n} & ≻\ldots ≻\;P_{R_{1}}^{n}\\ P_{IK_{2}}^{n} & ≻ & P_{IK_{2}-1}^{n} & ≻\ldots ≻\;P_{I_{1}}^{n} \end{array} \end{array}$$ (8)

If the matrix of transition probabilities $P_{R}^{n}$ and $P_{I}^{n}$ satisfy the assumption above, then $P_{R}^{n}$ and $P_{I}^{n}$ are *monotone matrices*[29]. Under this assumption, the higher the information value of the state of the current vertex the higher is the likelihood that the next state of this vertex will be of high information value, i.e., if $w_{I}^{n}(t)≻w_{I}^{n^{\prime }}(t)$, then $w_{I}^{n}(t)P_{I}^{n}≻w_{I}^{n^{\prime }}(t)P_{I}^{n^{\prime }}$. From (5), we know that probability vectors for information states of two vertices keep the relationship of stochastic dominance when no agent visits any of them. Obviously, if $w_{I}^{n}(t)≻w_{I}^{n^{\prime }}(t)$, then $w_{I}^{n}(t)F^{n}\geq w_{I}^{n^{\prime }}(t)F^{n^{\prime }}$, which means that a stochastically dominant information belief vector is likely to have a higher information value. The same is true that a stochastically dominant threat belief vector is likely to have a higher damage value. In particular, as the information state at a given vertex will reset to *I*_1_ when there is an agent visiting this vertex, the belief vector of information states (1,0,…,0) is stochastically dominated by the belief vector of any vertex which is not being visited, so the more recently visited vertex always has a lower expected information value.

To note, our monotonicity assumption is not a constraint that makes the information value (or the damage of the threat) increasing with time, but a model that the probability vector of the information (or threat) transition matrices satisfy the feature of stochastic dominance. We now provide a example of a 4-state Markov threat model at a vertex as follows:

$$\begin{array}{c} P_{R}=[\begin{array}{cccc} 0.8 & 0.1 & 0.1 & 0\\ 0.4 & 0.5 & 0.0 & 0.1\\ 0.2 & 0.1 & 0.6 & 0.1\\ 0 & 0.0 & 0.4 & 0.6 \end{array}] \end{array}$$

It can be seen that the Vectors of *P*_*R*_ satisfy the condition of Eq (8), i.e., *P*_*R*4_ ≻ *P*_*R*3_ ≻ *P*_*R*2_ ≻ *P*_*R*1_, where for *P*_*R*3_ ≻ *P*_*R*2_ as an example, the elements of *P*_*R*2_ and *P*_*R*3_ match the condition for stochastic dominance of Eq (7) as:

$$\begin{array}{c} \begin{array}{c} 0.1+0.6+0.1≧0.5+0.0+0.1\\ 0.6+0.1≧0.0+0.1\\ 0.1≧0.1 \end{array} \end{array}$$

For example, if the threat states at vertices *v*_1_ and *v*_2_ are respectively $w_{R}^{1}=[0.1,0.2,0.5,0.2]$ and $w_{R}^{2}=[0.2,0.4,0.3,0.1]$, i.e., $w_{R}^{1}≻w_{R}^{2}$. Then, *v*_1_ is likely to have a higher next threat state than *v*_2_. However, after a time step, it is possible that any threat state may switch to not only a higher state, but also a lower state.

Then given the monotonicity assumption, we can use the relationship between the belief states at different vertices in order to “predict” the belief state at an unvisited node. Hence, we can estimate the expected reward agents may get from one vertex of the graph when visiting it at a near future step. We denote a feasible policy of length *D* at time *t* as *π*_*D*_(*t*) = (*π*_*t*+1_,…, *π*_*t*+*D*_), which consists of *D* consecutive deterministic vertices/actions.

Here, we define the predictive heuristic as the predictive expected future reward $𝔼[\hat{ℛ}(\pi_{D}(t))]$ for policy *π*_*D*_(*t*), which is the aggregate of the expected reward of each step in *π*_*D*_(*t*) as:

$$\begin{array}{c} 𝔼\left[\hat{𝓡}\left(\pi_{D}\left(t\right)\right)\right]=\sum_{i=1}^{D}\gamma^{t}(\alpha {\hat{w}}_{I}^{\pi_{t+i}}\left(t+i\right)F^{\pi_{t+i}}-\left(1-\alpha \right){\hat{w}}_{R}^{\pi_{t+i}}\left(t+i\right)L^{\pi_{t+i}}) \end{array}$$ (9)

where, $\left[{\hat{w}}_{I}^{\pi_{t+i}}(t+i),{\hat{w}}_{R}^{\pi_{t+i}}(t+i)\right]$ is the predictive belief vector at the vertex *π*_*t*+*i*_ and time *t*+*i*. For the step *t*+1, we can get the predictive belief vector $\left[{\hat{w}}_{I}^{\pi_{t+1}}(t+1),{\hat{w}}_{R}^{\pi_{t+1}}(t+1)\right]$ by the current belief vector Ψ(*t*), current action *a*(*t*) and observations *θ*(*t*), i.e. $\Psi (t+1)=\delta \left(\Psi (t)|a_{t}^{*},\theta (t)\right)$, which is the belief vector at *t*+1 and obtained from Eq (5). For {*t*+2,…, *t*+*D*}, we get the predicted belief vector based on a transition which omits observations in Eq (5) as follows:

$$\begin{array}{c} \begin{array}{c} {\hat{w}}_{R}^{n}\left(\tau +1\right)=\begin{array}{c} {\hat{w}}_{R}^{n}\left(\tau \right)P_{R}^{n} \end{array}\\ {\hat{w}}_{I}^{n}\left(\tau +1\right)=\{\begin{array}{cc} P_{I1}^{n} & \text{if}\;v_{n}=\pi_{\tau }\\ {\hat{w}}_{I}^{n}\left(\tau \right)P_{I}^{n} & \text{if}\;v_{n}\neq \pi_{\tau } \end{array} \end{array} \end{array}$$ (10)

where *τ* = {*t*+1,…, *t*+*D*−1}.

Given the predictive heuristic and policies that looks ahead *D* time periods, the agent compares all feasible paths of length *D* and chooses the next location to visit according to the path that gives the highest predictive expected reward gained over that path. The details of how to use the heuristic in our online single-agent algorithm is presented in the next section.

#### The Online Algorithm

Based on the predictive heuristic, we propose an online algorithm for single-agent patrolling problem (Algorithm 1) in this section.

**Algorithm 1** Single-Agent Patrolling

**Require:** $\left\{P_{R}^{n}\right\}$: the Markov threat models

**Require:** $\left\{P_{I}^{n}\right\}$: the Markov information models

**Require:** Ψ(*t*): the belief state of current time step

**Require:** *o*(*t*): the observation at the current position

**Require:** *v*(*t*): current position.

**Ensure:** a*(*t*+1): next action of the agent

  ▷ *Step 0: get all feasible policies* Π_*D*_(*t*);

  ▷ *Step 1: computing best policy:*

1: **for** *π*_*D*_(*t*) ∈ Π_*D*_(*t*) **do**

  ▷ *Step 1.1: Get predictive belief state for next D steps:*

2:   Ψ(*t*+1) ← *δ*(Ψ(*t*)∣*v*_*t*_, *θ*(*t*))

3:   **for** *τ* ∈ {*t*+1,…, *t*+*D*−1} **do**

4:    **for** *v*_*n*_ ∈ *V* **do**

5:     ${\hat{w}}^{n}(\tau +1)\leftarrow \hat{\delta }\left({\hat{w}}^{n}(\tau )|\pi_{\tau }(\tau )\right)$

6:    **end for**

7:   **end for**

 ▷ *Step 1.2: Compute the predictive reward for π_D_(t):*

8:   $𝔼[\hat{ℛ}(\pi_{D}(t))]=\alpha w_{I}^{\pi_{t+i}}(t+i)F^{\pi_{t+i}}+\beta w_{R}^{\pi_{t+i}}(t+i)L^{\pi_{t+i}}$

 ▷ *Step 1.3: Compare π_D_(t) with the stored best policy:*

9:   **if** $𝔼[\hat{ℛ}(\pi_{D}(t))]>𝔼[\hat{ℛ}(\pi_{D}^{*}(t))]$ **then**

10:    $\pi_{D}^{*}(t)\leftarrow \pi_{D}(t)$

11:   **end if**

12: **end for**

 ▷ *Step 2:return the next action from the best policy* $\pi_{i}^{*}$

13: **return** $a^{*}(t+1)\leftarrow \pi_{t+1}^{*}$

First, we compute Π_*D*_(*t*), which is the set of all the feasible policies that start from current position *v*(*t*) (*step* 0), where we name the parameter *D* as the *maximum horizon*, i.e. the number of horizons we look ahead in the POMDP. Then, we compute the predictive expected reward for all the policies. For each policy, the belief state at *t*+1 is updated by the belief state, position and observations at *t* by Eq (5) (line 2) and the predictive belief state at {*t*+2,…, *t*+*D*} is computed by Eq (10) (line 3–7). Given this, we compute the predictive reward for the policy (line 8). Thus, the best policy is:

$$\begin{array}{c} \pi_{D}^{*}\left(t\right)=\underset{\pi_{D}\left(t\right)}{\text{arg max}}\;𝔼\left[\hat{𝓡}\left(\pi_{D}\left(t\right)\right)\right] \end{array}$$ (11)

The best next action here is computed as $a^{*}(t+1)=\pi_{t+1}^{*}$, which is the first action of best policy (line 13).

Having defined the online single-agent algorithm for our formulation of patrolling under uncertainty and threats, we extend it to compute policies for multi-agent problems next.

### Multi-Agent Patrolling

For multi-agent patrolling, we assume all the agents can share their collected observations with each other with full communication. Thus, team of agents may not only obtain more information about the environment, but each agent may also make decisions given observations are shared by other agents. Given this, we formulate the multi-agent patrolling problem as a *Multi-agent POMDP* (MPOMDP) and design an scalable online multi-agent algorithm to coordinate the actions of agents in their patrolling tasks.

A MPOMDP with complete communication can be reduced to a POMDP with a single centralised controller that takes joint actions and receives joint observations [30]. We now set up our problem of multi-agent patrolling in a graph as a POMDP ⟨ℳ,𝓢,𝓐,𝓞, *T*,Ω, *r*⟩ as follows.

- ℳ is the set of the agents.
- 𝓢 is the set of states. A *state* is defined as a tuple $\vec{s}=[\vec{v},(s_{R}^{1},\ldots ,s_{R}^{N}),(s_{I}^{1},\ldots ,s_{I}^{N})]\;\in \;𝓢$, where $\vec{v}$ is the current positions of agents, $s_{R}^{N}\;\in \;R^{n}$ and $s_{I}^{N}\;\in \;I^{n}$ are the threat and information states at vertex *v*_*n*_ ∈ *V*. We denote $\vec{s_{e}}\;\;=\;\;[(s_{R}^{1},\ldots ,s_{R}^{N}),(s_{I}^{1},\ldots ,s_{I}^{N})]\;\in \;{𝓢}_{e}$, as the state that captures the information and threat states at each position.
- 𝓐 is the set of all joint actions. The agents select adjacent vertices to visit as an *joint action*.
- 𝓞 is the set of joint observations. For current positions of the agents and the information and threat states of their current positions, we define a *joint observation* $o=\{\vec{v},\left\{o^{i}|\forall v_{i}\in \vec{v}\right\}\}\in O$, where $o^{i}=(s_{R}^{i},s_{I}^{i})$ is the observation of agent *i*.
- 𝓣 is the set of conditional transition probabilities. We assume that $\vec{v}$ is deterministic and only determined by the destinations of the joint movement of agents. $\vec{s_{e}}$ follows a discrete-time Markov process with $\prod_{n=1}^{N}K_{R}^{n}K_{I}^{n}$ states.
- Ω is the set of observation probabilities. As an observation $\vec{o}$ is directly a part of some states, the observation probability $\Omega (\vec{o}|\vec{s}{\;}^{\prime },\vec{a})=1$ if $\vec{o}$ is consistent with the corresponding part of $\vec{s}{\;}^{\prime }$ and $\Omega (\vec{o}|\vec{s}{\;}^{\prime },\vec{a})=0$ otherwise.
- *r*:𝓐 × 𝓞 → ℝ is a reward function. $r(\vec{a},\vec{o})$ is the sum of the reward obtained by the agents which associates to the joint action $\vec{a}$ and observation $\vec{o}$:
  $$\begin{array}{c} r\left(\vec{a},\vec{o}\right)=\sum_{v_{i}\in \vec{v}}(\alpha \frac{1}{n_{v_{i}}}f^{i}\left(s_{I}^{i}\right)-\left(1-\alpha \right)c^{i}\left(s_{R}^{i}\right)) \end{array}$$
  where *n*_*v*_*i*__ is the number of agents who are visiting *v*_*i*_.

The objective of the agents is then to choose the movement actions sequentially to maximize the total expected reward accumulated over *T* steps.

Then, we note that, while the state variable described in Eqs (2) and (3) can be used to express the belief vector of the environment states for a multi-agent POMDP, the joint action space of the POMDP is the Cartesian product of the action and observation spaces of the individual agents. However, in so doing, the size of the joint action space and joint observation space grows exponentially with the number of agents ∣ℳ∣, allowing only the smallest of problem instances to be solved. Instead, sequentially computing policies for individual agents as in our multi-agent algorithm avoids this problem of computing a joint policy for the team at the expense of solution quality. However, a bounded optimal of this multi-agent algorithm is guaranteed (we analyse this later).

Similar methods have been successfully used to solve multi-agent problems [3, 8]. As these formulations are different from our partially observable scenarios, a straightforward application of their methods is not possible. Hence, we consider how to sequentially compute policies for individual agents in partially observable problem using our online single-agent algorithm.

When sequentially computing policies for individual agents using our predictive heuristic, there implicitly exists an order in which the agents make actions; agent 1 completes *D* step actions of its best policy, agent 2 second, etc.. The expected future reward of a policy $\pi_{D}^{i}(t)$ of agent *i* is conditioned on position *v*_*i*_(*t*), belief vector Ψ(*t*) and the best policies of the previously computed policies of agents ℳ_−*i*_ = {1,…, *i*−1}.

The best online patrolling policy for agent *i* in a multi-agent setting is recursively defined as:

$$\begin{array}{c} \begin{array}{c} {\hat{\pi }}_{1}^{*}=\text{arg}\;{\text{max}}_{{\hat{\pi }}_{1}}{𝓡}^{\prime }(v_{1}\left(t\right),\Psi \left(t\right))\\ {\hat{\pi }}_{2}^{*}=\text{arg}\;{\text{max}}_{{\hat{\pi }}_{2}}{𝓡}^{\prime }(v_{2}\left(t\right),\Psi \left(t\right),{\hat{\pi }}_{1}^{*})\\ \;\vdots \\ {\hat{\pi }}_{i}^{*}=\text{arg}\;{\text{max}}_{{\hat{\pi }}_{i}}{𝓡}^{\prime }(v_{i}\left(t\right),\Psi \left(t\right),{\hat{\pi }}_{1}^{*},\ldots ,{\hat{\pi }}_{i-1}^{*}) \end{array} \end{array}$$ (12)

where we use ${\hat{\pi }}_{i}^{*}$ denotes the best policy of agent *i*.

To ensure the reward function only takes into account the marginal reward value, we need to exclude double counting. There are two types of double counting. First, *synchronous double* counting, which occurs when two agents patrol the same cluster within the same time step. In this case the reward for patrolling the vertex is received twice. Second, *asynchronous double counting*, which occurs when agent *i* decides to visit vertex *v*_*n*_ at *t*_1_, and there was an action of visiting *v*_*n*_ by agent *j* (*j* < *i*) at *t*_2_ (*t*_1_ < *t*_2_) during the *D* horizon i.e., the agent *j* will visit vertex *v*_*n*_ after agent *i*. For the situation where agent *j* visits vertex *v*_*n*_ before agent *i* (i.e. *t*_1_ ≥ *t*_2_), it has been accounted when calculating $𝔼[\hat{ℛ}(\pi_{D}(t))]$ in Eq (9).

Here, we show how to deal with the asynchronous double counting, i.e., agent *i* decides to visit vertex *v*_*n*_ at *t*_1_ and there was an action of visiting *v*_*n*_ by agent *j* (*j* < *i*) at *t*_2_ (*t*_1_ < *t*_2_) during the *D* horizon. Without loss of generality, we consider the situation that only *v*_*n*_ in $\pi_{D}^{i}(t)$ of agent *i* has been visited by agent *j*. If more than one agent of ℳ_−*i*_ = {1,…, *i*−1} has an action to visit *v*_*n*_, we assume the time *t*_2_ is nearest to *t*_1_ (only the nearest one needs to be taken into account and this can be deduced from the transition Eq (10)). Based on this assumption, we can see that the expected information reward of agent *j* for visiting vertex *v*_*n*_ is overestimated, as it is unaware that the *i* will reset the information at the time *t*_1_. Thus, we introduce a penalty $\hat{p}\in {\mathbb{R}}^{+}$ for agent *i* that compensates for the reduction of reward of agent *j*, as follows:

$$\begin{array}{c} {𝓡}_{i}^{\prime }(v_{i}\left(t\right),\Psi \left(t\right),{\hat{\pi }}_{1}^{*},\ldots ,{\hat{\pi }}_{i-1}^{*})=𝔼\left[\hat{𝓡}\left(\pi_{D}\left(t\right)\right)\right]-\hat{p} \end{array}$$ (13)

where $𝔼[\hat{ℛ}(\pi_{D}(t))]$ is the expected reward function defined in Eq (9), and $\hat{p}$ is the loss incurred by agent *j* that will visit the vertex *v*_*n*_ after *i*, which is defined as follows:

$$\begin{array}{c} \hat{p}={\hat{r}}_{\text{expected}}-{\hat{r}}_{\text{revised}} \end{array}$$ (14)

where the ${\hat{r}}_{\text{expected}}\in {\mathbb{R}}^{+}$ is the expected reward that agent *j* computes for visiting vertex *v*_*n*_ and the ${\hat{r}}_{\text{revised}}$ is the revised expected reward of agent *j* visiting vertex *v*_*n*_ as computed by agent *i* considering only its action. We define the *revised expected belief states* at vertex *v*_*n*_ and between time [*t*_1_+1,…, *t*_2_] are $\left\{{\tilde{w}}^{n}(t_{1}+1),\ldots ,{\tilde{w}}^{n}(t_{2})\right\}$, which are obtained by the transition Eq (10) based on the predictive belief state ${\hat{w}}^{n}(t_{1})$ and action *a*(*t*_1_) = *v*_*n*_. Then the *revised expected reward* is as follows:

$$\begin{array}{c} {\hat{r}}_{\text{revised}}=\gamma^{t_{2}}(\alpha {\tilde{w}}_{I}^{n}\left(t_{2}\right)F^{n}-\left(1-\alpha \right){\tilde{w}}_{R}^{n}\left(t_{2}\right)L^{n})\; \end{array}$$ (15)

Now, using the algorithm to compute the policy of length *D* as before, we obtain an action for each individual agent. A team action is formed by combining these individual actions. This team action is not optimal, as the policy of agent *i* is computed greedily with respect to the policies of agents ℳ_−*i*_. However, we can still bound the the performance guarantees compared with the policy obtained by searching the joint action space.

We use the theorem from [31] to obtain a bound on the value of the greedily selected policies:

**Theorem 2** *Let g:2^E^ → ℝ be a non-decreasing sub-modular set function. The greedy algorithm that iteratively selects the element e ∈ E that has the highest incremental value with respect to the previously chosen elements I ∈ E:*

$$\begin{array}{c} e=\underset{e\in E\setminus I}{\text{arg max}}\;g\left(e\cup I\right)-g\left(I\right) \end{array}$$ (16)

*until the resulting set I has the desired cardinality k, has an approximation bound* $\frac{g(I_{G})}{g(I^{*})}$ *at least* $1-{\left(\frac{k-1}{k}\right)}^{k}$, where I* ∈ E is the optimal subset of cardinality k that maximises f.

For the number of agents ∣ℳ∣ in our formulation, the approximation bound of the greedy algorithm is $1-{\left(\frac{|ℳ|-1}{|ℳ|}\right)}^{|ℳ|}$. It has been shown in [3] that this approximation bound is monotonically decreasing with ∣ℳ∣, and thus as, for ∣ℳ∣ → ∞, the multi-agent policy yields at least ≈ 63% of the reward obtained using the best policy that searches the joint policy space for ∣ℳ∣ agents.

Having formulated the problem and designed both single-agent and multi-agent algorithms, we will evaluate our methods in the next section.

## Empirical Evaluation

To empirically evaluate our approach, we applied it to 10 and 15 agents continuously patrolling in a large graph, which contains 350 vertices and 529 edges. The online computing time limit is 0.5s because agents must decide which locations to visit at each time step within that time limit. As the single-agent algorithm in the paper can be seen as a special case of the multi-agent algorithm, we just present the results of the multi-agent algorithm here. In the aforementioned graph, we simulated two scenarios:

- **Scenario A:** we use the same Markov information and threat models for every vertex in the graph;
- **Scenario B:** we apply 3 different information and threat models to different vertices in the graph.

Notice that for Scenario A the information and threat models at different locations are homogeneous. However, as these information and/or threat are non-stationary, the information/or threat state are various among these locations. We use Scenario A aiming to capture the situation where the locations in the environment hold same types of information and threat. For Scenario B, the information and threat models at different locations are heterogeneous, i.e., different locations in the environment may hold various types of information and threat.

We set the parameters in reward function (i.e., Eq 1) and value function (i.e., Eq 6) as: the weight parameter *α* = 0.33 and the discount factor *γ* = 0.9. More specifically, in Scenario A, we define the two Markov chains as follow:

$$\begin{array}{c} P_{R}=[\begin{array}{ccc} 0.9 & 0.1 & 0\\ 0.4 & 0.4 & 0.2\\ 0.0 & 0.2 & 0.8 \end{array}] \end{array}$$

$$\begin{array}{c} P_{I}=[\begin{array}{ccccc} 0.8 & 0.1 & 0.1 & 0 & 0\\ 0.2 & 0.7 & 0.0 & 0.1 & 0\\ 0.1 & 0.1 & 0.7 & 0.1 & 0\\ 0 & 0.0 & 0.1 & 0.8 & 0.1\\ 0 & 0 & 0.0 & 0.1 & 0.9 \end{array}] \end{array}$$

**Fig 2:**
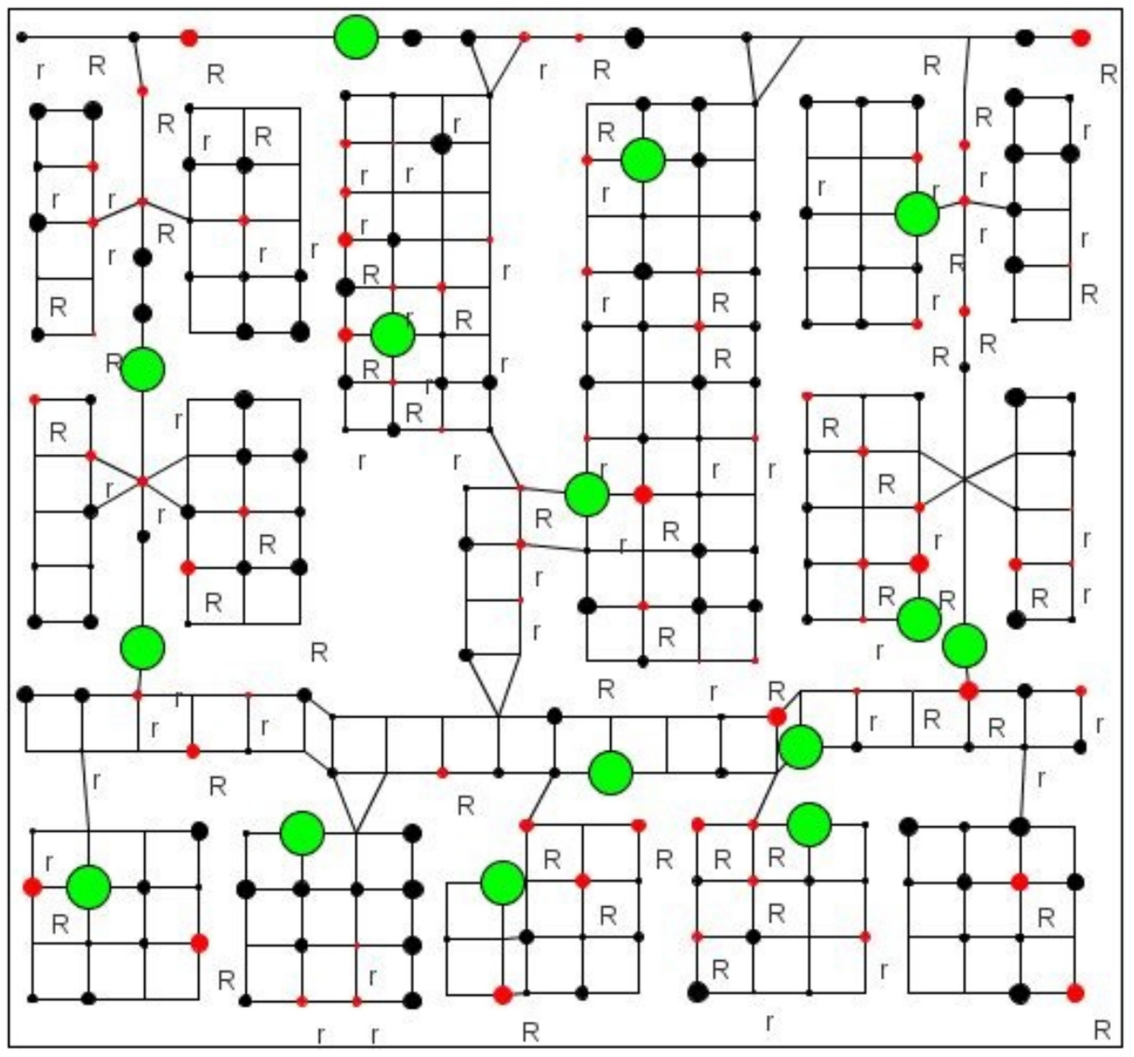
Scenario of 15 agents patrolling.

Here, the transition function *P*_*R*_ and *P*_*I*_ satisfies monotonic assumption of Eq (7). For example, the first two rows in *P*_*R*_ satisfy the constraints in Eq (8) that 0.9 ≥ 0.4, 0.9+0.1 ≥ 0.4+0.4 and 0.9+0.1+0.0 ≥ 0.4+0.4+0.2. The information and threat value vectors are respectively set as *F* = [0 1 2 3 4] and *L* = [0 1 2]. In Scenario B, we attribute several different Markov models to different vertices. A problem of 15 agents patrolling is shown in Fig 2, where the size of the circle of each location denotes the absolute value of instance reward of each vertex, the colour denotes its sign (black is positive and red is negative), the green circles are agents’ current locations and “R” and “r” in lower right of each vertex denotes the threat state of this vertex is “2” and “1” respectively.

For standard POMDP solvers such as POMCP, the size of the joint action space and joint observation space grow exponentially with the number of agents, which makes them are intractable for our multi-agent patrolling problem with large number of actions and observations. Hence, we benchmark against a random algorithm (Random) and a baseline algorithm (Baseline), and measure the total reward of the information value and the damage suffered of agents using them. Specifically,

- **Random** moves the agents to a random location adjacent to the agents’ current position.
- **Baseline** moves the agents to the adjacent location with the highest value in the next step. We assume the baseline algorithm sequentially computes policies for individual agents to avoid different agents selecting the same vertex, which is similar to PH-1.
- **PH-D** is our multi-agent patrolling algorithm, where *D* is the maximum horizon, i.e. the number of horizons we look ahead. We adjust maximum horizon *D* from the set {2,4,8} to investigate the extra computation involved for higher values of maximum horizon. We illustrated the results of our algorithms of different maximum horizon.

**Fig 3:**
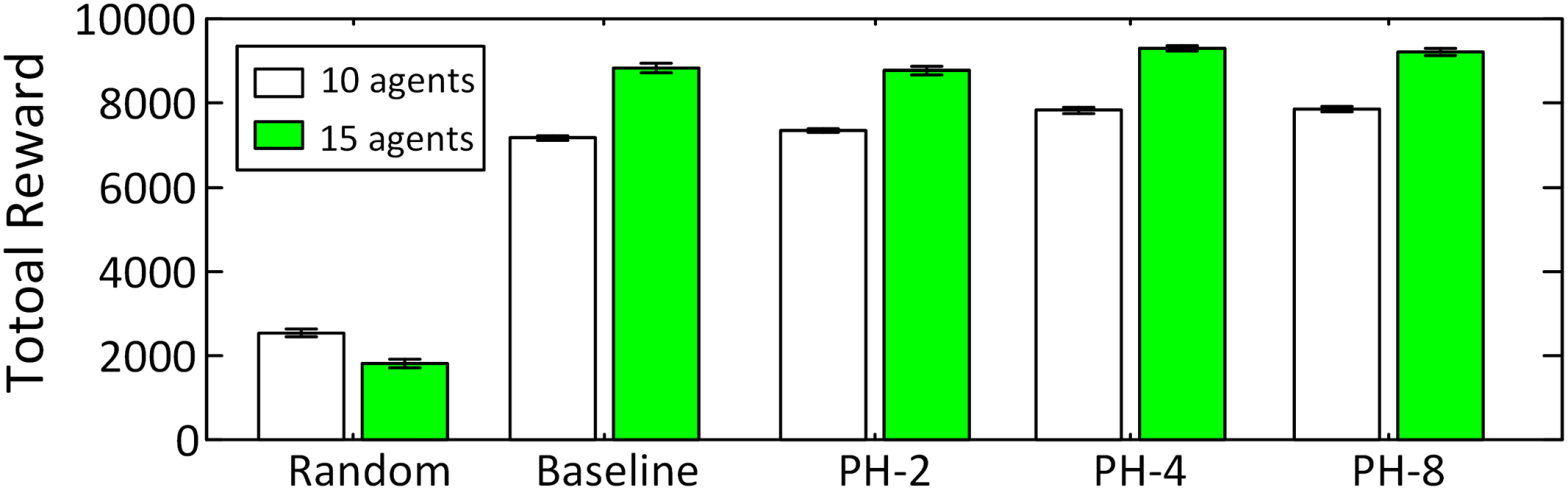
Rewards in Scenario A.

**Fig 4:**
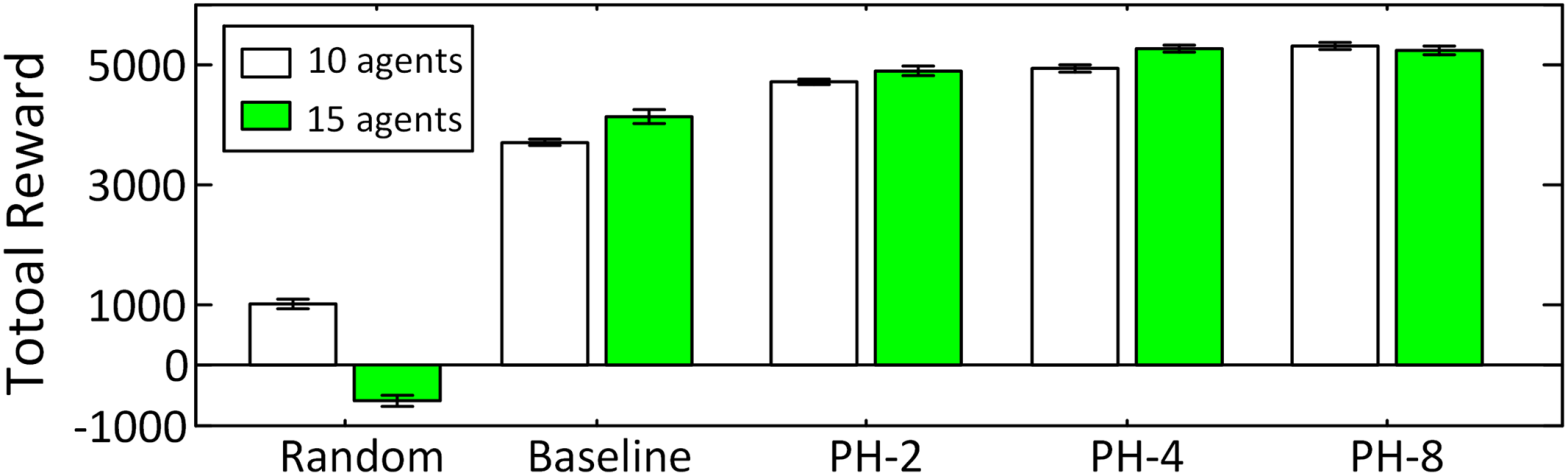
Rewards in Scenario B.

The initial locations of the agents are randomly distributed in the graph. Agents patrol continuously for 3000 time steps in the stochastically changing graph. For each scenario and each algorithm we ran 1000 rounds and plotted the results in Figs 3 and 4 where the error bars depict the 95% confidence intervals around the means. Non-overlapping error bars invalidate the null hypothesis with *α* = 0.05. In both scenarios, Random performs poorly and its total reward never reaches more than 30% of the reward obtained by the other two algorithms. In Scenario A, both PH-8 and Baseline perform well, and PH-8 outperforms than baseline algorithm by at least 5%. However, for the graph with different Markov models in Scenario B, our algorithm is significantly better than all the other algorithms, and PH-8 outperforms the baseline algorithm by more than 44% for 10 agents and by 21% for 15 agents. In addition, with different maximum horizon *D* from {2,4,8}, the reward obtained by PH-*D* increases with *D* as well as its computation time increases with *D* exponentially. For *D* > 8, the computing time for each step is out of our time limit for online decision making. Thus, we can conclude that the use of our predictive heuristic in Ph-*D* has a significant impact on performance and that *D* can be adjusted to trade-off between quality and computation time while still outperforming baseline algorithms.

## Conclusion

In this paper, we developed an online multi-agent patrolling algorithm for large partial observable and stochastic environment where the information are distributed with threats. Specifically, a predictive heuristic is defined to evaluate the policies of looking ahead several steps. For the multi-agent algorithm, we extended the sequential policy computation method for individual agents to deal with partially observable problems. We empirically showed that for 10 agents in a large graph, our algorithm outperforms the baseline algorithm by more than 44%. In our future work, on the one hand, as this is the first algorithm for patrolling with uncertainty and threats, we plan to devise a better heuristic and algorithms that provide theoretical performance guarantees in our future work. One the other hand, as our formulation is a basic model of UAVs patrolling under uncertainty and threats, we will consider that the communication system of the agents may locally break down by suffering from harms or some agents may get destroyed due to cumulated harms.

## Supporting Information

S1 Video Video to show the simulation of 15 agents patrolling problem. (MP4)

S1 Code Java code to implement the scenarios and computations in the paper. (ZIP)
